# A novel purified *Lactobacillus acidophilus* 20079 exopolysaccharide, LA-EPS-20079, molecularly regulates both apoptotic and NF-κB inflammatory pathways in human colon cancer

**DOI:** 10.1186/s12934-018-0877-z

**Published:** 2018-02-21

**Authors:** Nehal M. El-Deeb, Abdelrahman M. Yassin, Lamiaa A. Al-Madboly, Amr El-Hawiet

**Affiliations:** 1 Biopharmacetical Product Research Department, Genetic Engineering and Biotechnology Research Institute, City of Scientific Research and Technology Applications, New Borg El-Arab City, 21934 Alexandria Egypt; 2 Department of Pharmaceutical Microbiology, Faculty of Pharmacy, Tanta University, Tanta, 31527 Egypt; 3 Department of Pharmacognosy, Faculty of Pharmacy, Alexandria University, Alexandria, Egypt

**Keywords:** LAB, NFκB pathway, Oligosaccharides, T cells immunophenotyping, Colon cancer

## Abstract

**Background:**

The direct link between inflammatory bowel diseases and colorectal cancer is well documented. Previous studies have reported that some lactic acid bacterial strains could inhibit colon cancer progression however; the exact molecules involved have not yet been identified. So, in the current study, we illustrated the tumor suppressive effects of the newly identified *Lactobacillus acidophilus* DSMZ 20079 cell-free pentasaccharide against colon cancer cells. The chemical structure of the purified pentasaccharide was investigated by MALDI-TOF mass spectrum, 1D and 2D Nuclear Magnetic Resonance (NMR). The anticancer potentiality of the purified pentasaccharide against both Human colon cancer (CaCo-2) and Human breast cancer (MCF7) cell lines with its safety usage pattern were evaluated using cytotoxicity, annexin V quantification and BrdU incorporation assays. Also, the immunomodulatory effects of the identified compound were quantified on both LPS-induced PBMC cell model and cancer cells with monitoring the immunophenotyping of T and dendritic cell surface marker. At molecular level, the alteration in gene expression of both inflammatory and apoptotic pathways were quantified upon pentasaccharide-cellular treatment by RTqPCR.

**Results:**

The obtained data of the spectroscopic analysis, confirmed the structure of the newly extracted pentasaccharide; (LA-EPS-20079) to be: α-d-Glc (1→2)][α-l-Fuc(1→4)] α-d-GlcA(1→2) α-d-GlcA(1→2) α-d-GlcA. This pentasaccharide, recorded safe dose on normal mammalian cells ranged from 2 to 5 mg/ml with cancer cells selectivity index, ranged of 1.96–51.3. Upon CaCo-2 cell treatment with the non-toxic dose of LA-EPS-20079, the inhibition percentage in CaCo-2 cellular viability, reached 80.65 with an increase in the ratio of the apoptotic cells in sub-G0/G1 cell cycle phase. Also, this pentasaccharide showed potentialities to up-regulate the expression of IKbα, P53 and TGF genes.

**Conclusion:**

The anticancer potentialities of LA-EPS-20079 oligosaccharides against human colon cancer represented through its regulatory effects on both apoptotic and NF-κB inflammatory pathways.

**Electronic supplementary material:**

The online version of this article (10.1186/s12934-018-0877-z) contains supplementary material, which is available to authorized users.

## Background

Cancer, is one of the major causes of morbidity and mortality worldwide [1]. Cancer cells, are altered self-cells that have escaped the normal growth regulatory mechanisms. Under normal conditions, a balance is maintained between the rate of cell division and death keeping the number of a certain cell type constant. Cancer cells are characterized by the presence of multiple cell physiology alterations including; self-sufficiency in growth signals, resistance to growth-inhibitory signals and programmed cell death, limitless replicative potential, sustained angiogenesis and metastasis [2].

The available approaches for cancer treatment include chemotherapy, surgery and radiation. Chemotherapy represents the main choice of treatment in most cases. However, the majority of conventional chemotherapeutic agents, targeting actively dividing cells, do not show selective toxicity towards cancer cells leading to healthy cell damage. Moreover, cancer cells might develop resistance to conventional chemotherapeutic agents through various mechanisms, such as, increased expression of drug detoxifying enzymes and drug transporters and increased ability to repair DNA defects in cellular machinery that mediate apoptosis [3]. Therefore, there is a pressing demand for targeted therapies that are capable of killing cancer cells selectively without affecting normal healthy cells or at least to act as adjuvants to lower the therapeutic doses and increase efficiency of conventional anticancer drugs. Synthesis of exopolysaccharides by lactic acid bacteria (LAB) is a well-known phenomenon which exists as a cell-bound EPS, adhering closely to the bacterial surface, or releases EPS into the surrounding medium [4].

Lactic acid bacteria (LAB) is one of the well-known bacteria for synthesizing EPS which starts as a cell bound EPS adhering to the bacterial surface followed by its release in the surrounding medium. They are believed to play a significant role in colonization of LAB in the intestinal mucosa [4, 5]. Some EPSs, extracted from LAB, show multiple health benefits by acting as anti-tumor, anti-biofilm agents, in addition to their ability to enhance immunity and their antioxidant properties which have particularly received significant attention by many research groups. For example, Joo Seo et al. [6] reported the extraction and purification of EPSs from *Lactobacillus plantarum* YML009 with a very potent antioxidant activity. Another example is the EPS extracted from *Lactobacillus plantarum* 70810 which shows a very promising antiproliferative effects against Hepatocellular carcinoma cell line (HepG-2) [7].

In this study, we purified and identified a novel EPS from *Lactobacillus acidophilus* DSMZ 20079, and evaluated their selective cytotoxic effect against Human colon cancer cells in parallel with their immunomodulatory behavior. In addition, the possible mechanisms of the anticancer activities of the extracted EPS were studied on both cellular and molecular levels. The current study is considered as the first report that explained the inhibitory effects of *Lactobacillus acidophilus* DSMZ 20079 EPS against cancer cells.

## Methods

### Mammalian cell lines

The non-cancerous cells; African Green Monkey Kidney cells (VERO), Canine Kidney cells (MDCK) and Syrian Hamster Kidney cells (BHK), were cultured on DMEM media and Human Peripheral Blood Mononuclear Cells (PBMC) were cultured on RBMI media.

The cancerous cell lines; Human breast cancer cells (MCF7) were cultured on RBMI media and Human Colon cancer cells (CaCo-2), were cultured on DMEM media. All used media, were supplemented with 200 mM l-glutamine and 10% fetal bovine serum (FBS; Gibco-BRL) and 1% penicillin/streptomycin. PBMCs were isolated by Gradient centrifugation, as reported by Lohr et al. [8].

### Bacterial strains and culture conditions

For inoculation, aliquot of 1 ml (4.0 × 10^8^ CFU/ml) of *L. acidophilus* DSMZ 20079 overnight culture, was inoculated in De Man-Rogosa-Sharpe (MRS) broth and then incubated at 37 °C, under aerobic conditions for 24–48 h. At the early stationary growth phase (26 h incubation, Additional file 1: Figure S1), the bacterial culture was centrifuged at 10,000 rpm at 2 °C for 30 min and culture supernatant was separated carefully and filtered through a 0.22 µm pore-size filter.

### Production, purification and identification of exopolysaccharides

For exopolysaccharide (EPS) extraction, at the end of incubation time, the culture supernatant was treated with 10% trichloroacetic acid (1:1), and then centrifuged at 10,000 rpm at 2 °C for 30 min. The clear supernatant was subjected to 3 successive 3 volume absolute alcohol extraction. At the end of extraction time; the EPS, were collected by centrifuged at 10,000 rpm at 2 °C for 30 min. The obtained ethanol soluble EPS were recovered in a rotary evaporator at 40 °C and stored at 4 °C until the time of analysis. The extracted EPS were dialyzed against ddH_2_O over 5 days using dialysis membrane a having MWCO 1000 Da (thermo fisher scientific). The purified using DEAE-cellulose column as described by Sheng et al. [9]. The structure of this compound was elucidated using mass spectrometry, a combination of 1D (^1^H and ^13^C) and 2D NMR spectroscopy,

#### A-mass spectrometry

The test polysaccharide was mixed at a ratio of 1:1 with water. About 2 µl of the sample was loaded on a 800 µm Anchorchip target plate (Bruker Daltonic, Germany). Sample was analyzed with an Autoflex III matrix assisted laser desorption ionization-time-of-fligh (MALDI-TOF/TOF) mass spectrometer (Bruker-Daltonics) equipped with a nitrogen laser emits at 337 nm and a 3 ns pulse width). Automated analysis of mass data was performed using Flex Analysis software (Bruker-Daltonics). The laser was used at a frequency of 200 Hz and the power was adjusted manually until the optimal ratio of the signal to the noise was obtained, which usually ranges between 40 and 60%. Each accumulated spectrum resulted in at least 4000 laser shots [10].

#### B-NMR identification

1D (^1^H- and ^13^C)-NMR spectra were measured in DMSO-*d6* with a JEOL ECA 500 NMR (JEOL Ltd., Tokyo, Japan) spectrometer, at 500 and 125 MHz, respectively. The chemical shift (δ) was reported in parts per million (ppm). The *J* value was reported in Hz. For 2D-NMR spectra identification, twenty milligrams of the freeze-dried EPS was dissolved in D2O (0.5 ml, 99.9%). Twenty milligrams of EPS was dissolved in D2O (0.5 mL, 99.9%), freeze-dried, and redissolved in D2O (0.5 ml). The spectra were measured in an NMR tube (5 mm diameter) at 27 °C [11].

### Cytotoxicity assay (safety pattern assay)

The safety usage pattern of the extracted EPS was tested on different normal and non-cancerous cell; PBMC, VERO, MDCK and BHK, using neutral red assay protocol. Briefly, about 100 µl of each serially diluted EPS was incubated with pre-cultured cell (seeding intensity 6 × 10^4^ cell/ml), on 96-well plates. After 48 h, in order to assess the nontoxic dose, dose response studies were quantified using neutral red assay, as described by Borenfreund and Puerner [12] which is based on the accumulation of the neutral red dye, in the lysosomes of viable, uninjured cells. The dye intensity measurements were referenced to that given by the control cells; cells without any treatments. Each measurement was repeated three times and the means were calculated as well as the standard deviation (SD), using graphpad prism 7 software.

### Anticancer activities of *L. acidophilus* EPS

The anticancer activities of *L. acidophilus* EPS on both CaCo-2 and MCF7 cells were monitored using phase contrast inverted microscope and quantified, using neutral red assay as previously described.

### Selectivity index (SI)

Cancer cell selectivity index of the recovered polysaccharides, was calculated as the previous report of Koch et al. [13] with a minor modification; SI = IC50nc/IC50cc, where IC50nc refers to the IC50 value of the tested compound on normal cells while; IC50cc refers to the IC50 of the tested compound on cancer cell line.

### Cell proliferation assay

Cell proliferation, in response to treatments was assayed using the measurements of 5-bromo-2-deoxyuridine (BrdU) that incorporated into cellular DNA, during cell proliferation, using cell Proliferation ELISA BrdU Kit (Roche Applied Science), according to the kit manufacturer’s protocol. Each measurement was repeated three times and the means were calculated as well as the standard deviation (SD), using graphpad prism 7 software.

### Apoptosis assessment by annexin V assay and acridine orange/ethidium bromide stain

Annexin V apoptosis detection assay, based on the observation that soon after initiating apoptosis; most cell types translocate the membrane phospholipid phosphatidylserine (PS) from the inner face of the plasma membrane to the cell surface. This translocated PS was quantified using annexin V Apoptosis Detection (e Bioscience). After cellular (MRC5, PBMC and CaCO-2) treatment, the translocated PS was quantified using annexin V apoptosis detection kit.

### Acridine orange (AO/EB)

Acridine orange is a vital dye; it could stain both live and dead cells while; ethidium bromide could stain only cells that have lost membrane integrity. After treatment, About 25 µl of cell suspension (0.5 × 10^6^ cells/ml) was incubated with 1 µl of AO/EB solution. Using a fluorescence microscope, 10 µl of cell suspension was examined onto a microscopic slide [14].

### Cell cycle analysis by flow cytometry

The alterations in cell cycle pattern were determined by using flow cytometry and according to Léonce et al. [15]. In this assay, propidium iodide (PI) can be employed to discriminate living cells from dead cells, or for cell cycle analysis; cell cycle analysis is based on the stoichiometric binding of PI to intracellular DNA. After 48 h of cellular treatment, cells were washed with warm PBS and collected by trypsinization. The collected cells (about 2 × 10^5^ cells/ml) were then re-suspended in warm PBS, fixed with about 4 ml ice cold absolute ethanol and then stained for 30 min with 0.5 mL of warm PI solution (7 ml of PI solution consists of 0.35 ml of PI solution (1 mg/ml), 0.7 ml RNase A solution (1 mg/ml), and 6 ml of PBS). The all samples were kept on ice until flow cytometric analysis.

### Immunomodulatory activities of *L. acidophilus* EPS

#### Immunophenotyping analysis of T and dendritic cell surface marker using flow cytometry

About 2 × 10^5^ cells/ml of PBMC was seeded in RPMI medium, in a rounded bottom 96-well plate for 24 h. At the end of incubation, cells were treated with the non-toxic dose of purified pentasaccharides for 48 h. After treatment, cells were stained with the following anti-Human monoclonal antibodies; phycoerythrin (PE)-conjugated anti-CD8, allophycocyanin (APC) conjugated anti-CD4, phycoerythrin (PE)-conjugated anti-CD123 and allophycocyanin (APC) conjugated anti-CD11b, in the dark for 30 min at 4 °C. The percentages of CD4+ T cells, CD8+ T cells, CD11b Dendritic Cells (DCs) and CD 123 Dendritic DCs cells were determined by flow cytometry.

#### Quantification of the induced reactive oxygen species in both colon cancer and epithelial cells during pentasaccharide treatment

The intracellular-induced reactive oxygen species (ROS) was quantified in both CaCo-2 cells and normal epithelial cells (MRC5), using the fluorescence membrane-permeable probe 2,7-dichlorofluorescein diacetate (DCFH-DA) (Molecular Probes, Sigma-Aldrich). The fluorescence probe was added after cellular treatment at a final concentration of 20 μM according to Sawada et al. [16] and El-Adawi et al. [17]. At the end of incubation, the obtained fluorescence was quantified, using flow cytometry.

#### Quantification of the induced cytokines and ROS in LPS-induced PBMC cells

About 2 × 10^5^ cells/ml of PBMCs were suspended in RPMI medium and seeded into a rounded bottom, 96-well plate for 24 h. At the end of incubation, the inflammatory model was induced by stimulating PBMC with 100 µl of *E. coli* LPS (10 mg/ml) for 24 h in the presence or absence of the treatment.

At the end of incubation, the levels of IL-2 & 8 were quantitatively measured using Thermo Scientific^®^ Human IL-2 & 8 ELISA Kit, according to the manual instruction. Each measurement was repeated three times and the means were calculated as well as the standard deviation (SD), using graphpad prism 7 software. Also, TNF-α gene expression was quantified, using RTqPCR.

The intracellular induced ROS was assessed, using the fluorescent membrane permeable probe 2,7-dichlorofluorescein diacetate (DCFH-DA) (Molecular Probes, Sigma-Aldrich) as described above.

### RNA extraction and real-time RT-PCR analyses for TNF-α-gene expression

After treatment, the total RNA was extracted from PBMC cells, using Thermo Scientific MagJET Whole Blood RNA Kit, according to manufacturer’s protocol. By using Thermo Scientific cDNA Synthesis Kit, cDNA was synthesized. The quantitative real-time PCR was performed using the Maxima SYBR Green/ROX qPCR Master Mix using forward and reverse primer (Table 1) and SYBR green PCR master mix, according to the manufactured protocol. The thermal cycling protocol of RT was as follows: 50 °C for 2 min, 95 °C for 15 min 40 cycles of (15 s at 94 °C, 30 s at 50 °C and 30 s at 72 °C). The TNF-α genes were normalized with reference gene β-actin.

**Table 1:** The list of primer used in gene expression analysis

Primers	Sequence
P53-F	5′-AACGGTACTCCGCCACC-3′
P53-R	5′-CGTGTCACCGTCGTGGA-3′
IkappaB-α F	5′-CATGAAGAGAAGACACTGACCATGGAA-3′
IkappaB-α R	5′-TGGATAGAGGCTAAGTGTAGACACG-3′
TNF-α F	5′-TTC TGT CTA CTG AAC TTC GGG GTG ATG GGT CC-3′
TNF-α R	5′-GTA TGA GAT AGC AAA TCG GCT GAC GGT GTG GG-3′
TGF F	5′-CAAGGGCTACCATGCCAACT-3′
TGF R	5′-AGGGCCAGGACCTTGCTG-3′

#### The molecular mode of action of *L. acidophilus* EPS on CaCo-2 cells

The activities of EPS on regulating the expression of some genes as TGF, IKaB and P53 of CaCo-2 were quantified by RTqPCR. Briefly; CaCo-2 cells were treated with the resulted nontoxic concentration of EPS, for 24 h at the previous incubation conditions. After incubation, the total RNA was extracted, the first cDNA strand was synthesized and RTqPCR was performed as previously described. The used primers were listed in Table 2. Each experiment was repeated three times and the means were calculated, as well as, the mean Cq using CFX Manager software (CFX96 Touch™ System, biorad).

**Table 2:** Quantification of TGF, IKaB and P53 gene expression in Caco2 cells upon *L. acidophilus* LA-EPS-20079 treatment

Target	Sample	Mean Cq	Normalized expression	Regulation	Compared to regulation threshold
Actin	Control	31.01			No change
Actin	LAB	31.13			No change
IKaB	Control	33.72	0.15309	1.00000	No change
IKaB	LAB	25.51	49.21789	321.50671	Up regulated
P53	Control	42.52	0.00034	1.00000	No change
P53	LAB	37.12	0.01567	45.83160	Up regulated
TGF	Control	39.50	0.00277	1.00000	No change
TGF	LAB	9.72	2,792,763.65222	1,007,436,556.06075	Up regulated

### Statistical analysis

Statistical analysis was performed, using graphpad prism 6 software. Statistical differences in multiple groups were determined, by one-way ANOVA followed by multiple mean comparisons in Duncan’s test. The numerical data of the all tests are presented as mean standard deviation, and p ≤ 0.05 was considered statistically significant.

## Results

### Nuclear magnetic resonance and structural analysis of LA-EPS-20079 pentasaccharide

The ^1^H-NMR spectrum of LA-EPS-20079, showed the presence of five anomeric proton signals, at (5.21, 5.14, 5.08, 4.55 and 4.68 ppm), correlated with five carbon resonances, in the ^1^H-^13^C HSQC spectrum at (92.18, 96.00, 95.39, 100.28 and 96.00 ppm, respectively), indicating that LA-EPS-20079, is a pentasaccharide. The characteristic chemical shifts of 1.27 ppm in ^1^H-NMR spectrum and 16.24 ppm in ^13^C-NMR spectrum resonances was assigned for H6 and C6, respectively, of a 6-deoxyhexose which was assigned as fucose. The MALDI-TOF mass spectrum, in the positive mode, of LA-EPS-20079, showed a single peak at m/z 877.109, corresponding to a compound of molecular formula, C_30_H_46_O_28_Na that was attributed to the pseudomolecular ion [M + Na]^+^. According to the above analysis, LA-EPS-20079 is confirmed to be a pentasaccharide composed of three glucuronic acid, one glucose and one fucose residues.

The structural analysis of LA-EPS-20079 was performed, using the ‘determine structure’ module of the CASPER program by submitting selected unassigned ^1^H and ^13^C NMR data. The structural information was given as follows: three D-GlcAp, one l-fucose and one d-Glc, where all the linkage positions were marked as unknown. Five ^3^J_H1,H2_ of 2–7 Hz and five ^1^J_C1,H1_ > 169 Hz were used. The calculations performed by CASPER, resulted in a list of ten possible structures. A noticeable score difference was observed for the structure ranked in the first position and the rest of structures. These structures were ranked according to the deviation between predicted and experimental ^1^H and ^13^C chemical shifts. All the CASPER reports with the chemical shift assignments were shown in the Additional file 1: Figure S1.

**Fig. 1:**
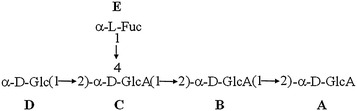
The ordering of the five residues in LA-EPS-20079 structure

Based on CASPER calculations, LA-EPS-20079 residues were named A–E in order of decreasing chemical shifts (Fig. 1) values of their anomeric protons. Residues A, B and C are assigned to glucuronic acid residues while residue D and E are assigned to glucosyl and fucosyl residues, respectively. The anomeric centers configurations were assigned based on the ^1^J_C1,H1_ couplings which showed couplings of 170–175 Hz for A–E residues confirming that all are α-linked as predicted by CASPER.

The complete assignment of ^1^H and ^13^C chemical shifts and the inter-residue connectivity of the monosaccharides residues of LA-EPS-20079 were further confirmed with the aid of two-dimensional COSY, HMBC, HSQC and NOESY experiments as indicated in Table 1. Combination of the structural analysis as presented above together with comparison with the previously published ^1^H-and ^13^C-NMR data (18, 19, 20, 21, 22) allowed LA-EPS-20079 to be formulated as α-d-Glc(1→2)][α-l-Fuc(1→4)] α-d-GlcA(1→2) α-d-GlcA(1→2) α-d-GlcA. It is worth mentioning this is the first report of this pentasaccharide from natural source.

### Safety pattern of *L.* acidophilus LA-EPS-20079 on non-cancerous cells

**Fig. 2:**
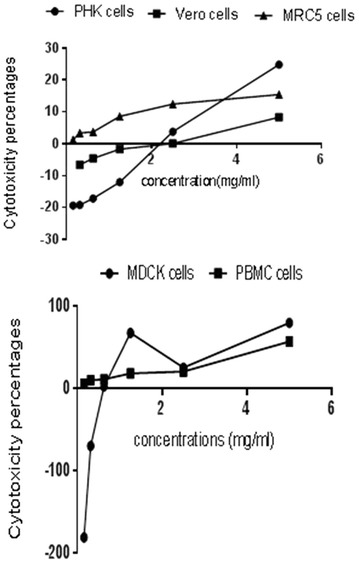
Cytotoxicity assay of *L. acidophilus* pentasaccharide (LA-EPS-20079) on various mammalian noncancerous cells

Using neutral red assay protocol, the safety pattern of LA-EPS-20079 was scanned on various non-cancerous and normal cells; MDCK, Vero, MRC5, PHK and PBMC (Fig. 2). The obtained data clarified that; LA-EPS-20079 safe dose ranged, from 2 to 5 mg/ml with ranged IC50 value of 2.61–0.68 µg/ml. Both Canine Kidney (MDCK) and PBMC were the most sensitive cells to the purified pentasaccharide and recorded IC50 values 2.61 and 4.89 mg/ml (p < 0.0001); respectively, while; Green Monkey kidney (VERO) and Human Lung Fibroblast cells (MRC5) were the most tolerant cells to the treatment. In addition, comparing with the controlled non treated cells, the proliferation rates of certain cells; MDCK, PHK and vero, increased upon LA-EPS-20079-treatment at doses ranged from 1.25 to 0.312 mg/ml, with increased proliferation percentages from 1.6 to 70.1.

### Anticancer activities of LA-EPS-20079 on both MCF7 and CaCo-2 cells

**Fig. 3:**
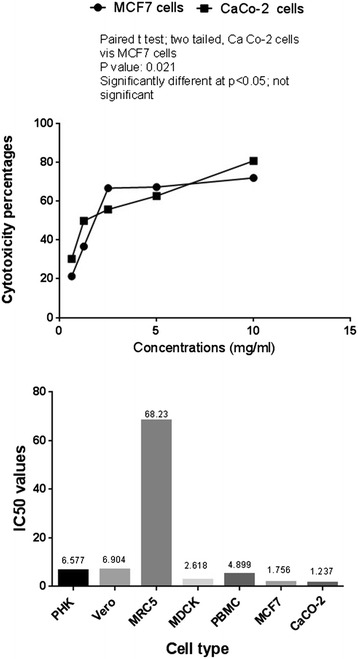
Anticancer activities of *L. acidophilus* oligosaccharides on Human breast and colon cancer cells

**Fig. 4:**
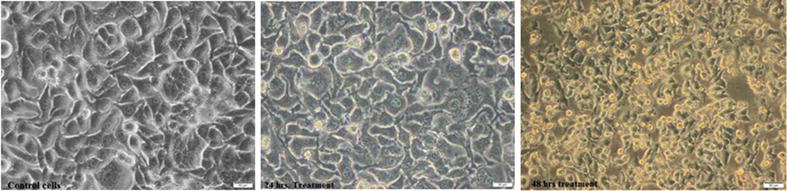
The morphological changes of caco2 cells during treatment for 24 and 48 h

The anticancer activities of the purified pentasaccharide against both CaCo-2 and MCF7 cells were quantified using neutral red and BRDU proliferation assays (Fig. 3). After 48 h of treatment, cellular viability inhibition percentage (neutral red quantification results), of MCF7 and CaCo-2-treated cells reached 71.86 and 80.65; respectively with 78.95 and 87.27106 percentage of inhibition, in cellular proliferation (BRDU cellular incorporation); significantly different from the non-treated cells (p < 0.05). By comparing the treatment of IC50 values (2.61 mg/ml to 0.68 µg/ml), on noncancerous cells with that on the cancer cells (1.336 mg/ml); LA-EPS-20079, exhibited significant cancer cell selectivity index, ranged from 51.3 to 1.96. In addition, the morphological characters of the treated cancer cells that were examined under the phase contrast microscope. (Figure 4) showed the appearance of undergoing apoptotic cells that characterized by cellular rounding up, shrinkage, membrane blebbing and loss of cell adhesion.

### Apoptosis assessment using annexin V assay and Acridine orange/ethidium bromide dual staining assay

**Fig. 5:**
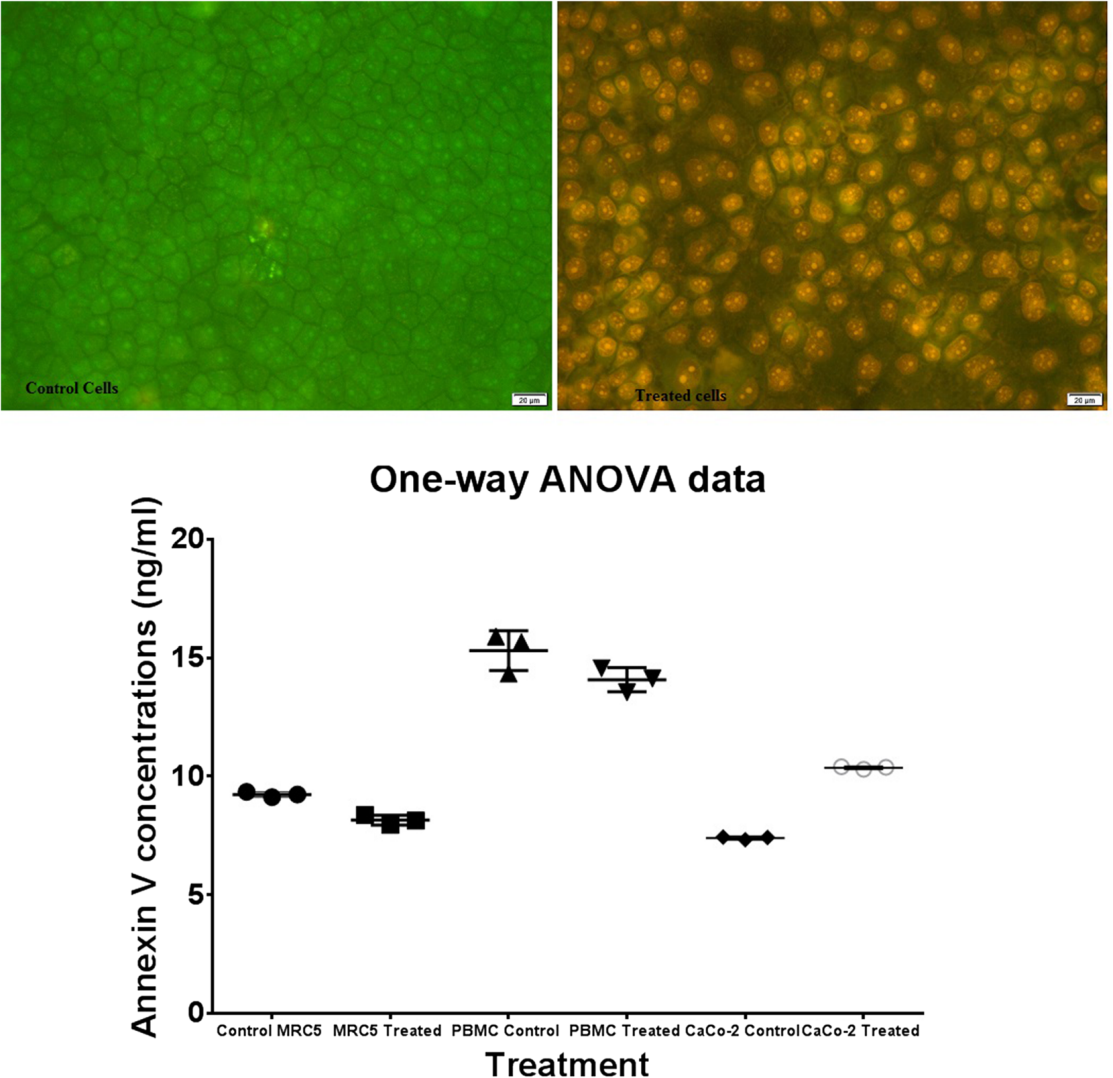
Apoptosis assessment using acridine orange/ethidium bromide dual stain and annexin V assay. The photomicrographs of apoptosis detected by acridine orange/ethidium bromide dual stain in human colon cancer cells. Total endogenous annexin V quantitatively detection with the ELISA

Annexin V is known to bind to the phosphatidylserine-exposing apoptotic cell and could inhibit the procoagulant and proinflammatory activities of the dying cell. Total endogenous annexin V was quantitatively detected by ELISA. The obtained results indicated that a cellular apoptosis CaCo-2 treated cell was induced after 24 h. of treatment with the purified pentasaccharide, as shown in (Fig. 5). The concentration of annexin V, significantly increased from 7.422 ng/ml in the control cells to 10.38 ng/ml, in treated ones (p ≤ 0.0001). In contrast, annexin V concentrations of treated normal cells (PBMC and MRC5), significantly decreased from 15.30 to 14.08 ng/ml (in the non-treated cells) and 9.231 to 8.150 ng/ml; respectively, compared with the non-treated cells.

On the other hand, acridine orange/ethidium bromide (AO/EB) staining was used to visualize nuclear changes during cellular apoptosis, via fluorescence microscope.

In the case of living cells, nuclei appeared with normal green staining with green chromatin and they showed organized structures, while, in the early apoptotic stage, cells showed condensed or fragmented chromatin (green or orange). However, during late apoptotic or necrotic stages, cells have exhibited similar normal nuclei staining, as live cells, but with orange chromatin. Upon cellular treatment with the pentasaccharide, the treated cells appeared with an early apoptotic feature as orange stained multinucleated cells (Fig. 5).

### Cell cycle analysis

**Fig. 6:**
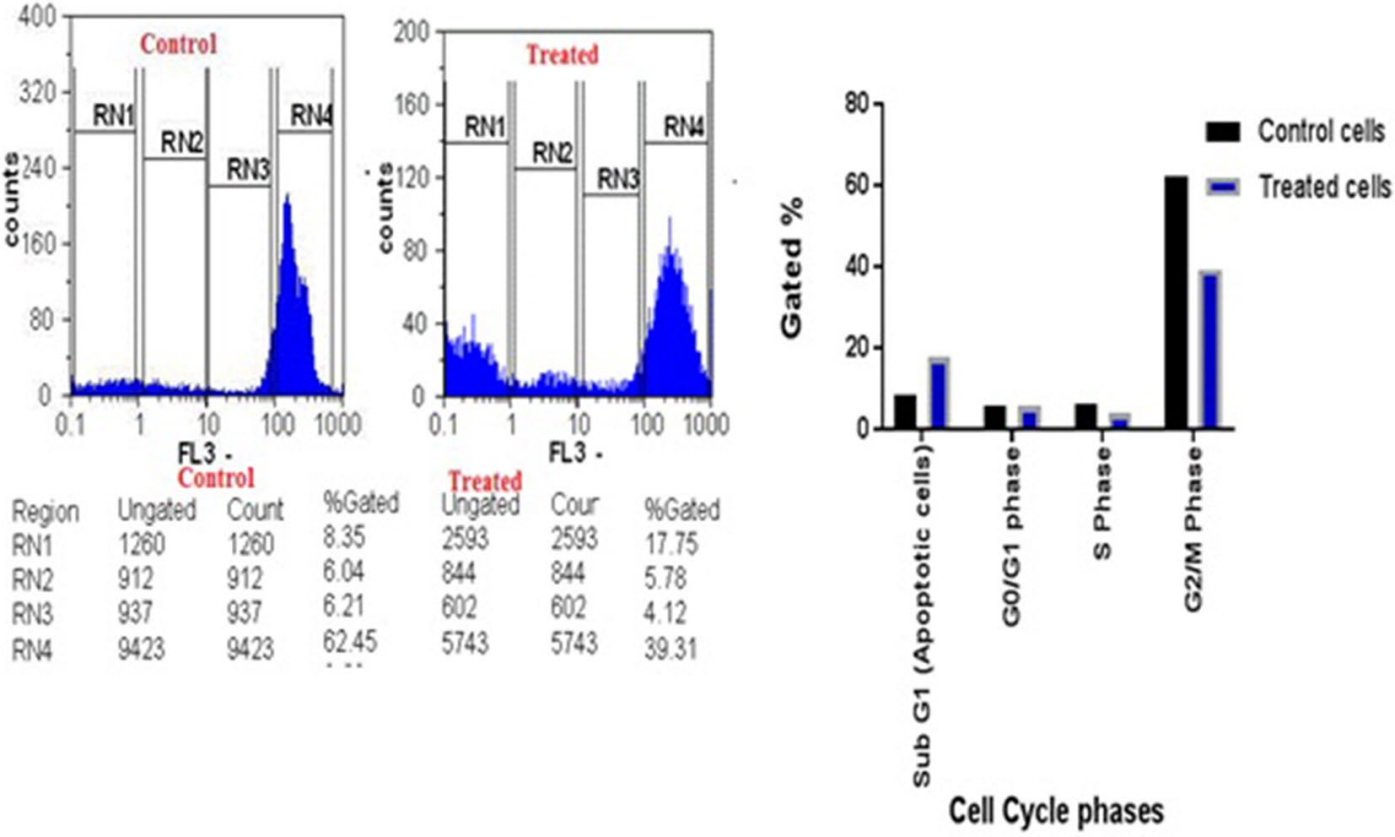
Effect of LA-EPS-20079 treatment on the regulation of cell cycle distributions of colon cancer cells. The distribution and percentage of cells in pre-phase, G1, S and G2/M phase of the cell cycle are indicated in the graph presented

The flow cytometric analysis for the cell cycle pattern of the treated cells is shown in Fig. 6. After treatment, the population percentages in sub-G0/G1 that represented the apoptotic cells were increased significantly in CaCo-2 treated cells to reach 17.75%, in comparison to the non-treated control cells. Both G1 and S cellular populations were slightly decreased, while, G2/M population percentages were dramatically decreased from 62.45 to 39.31% after treatment.

### Immunomodulatory activities of *L. acidophilus* EPS

#### Immunophenotyping analysis of T and dendritic cell surface marker using flow cytometry

**Fig. 7:**
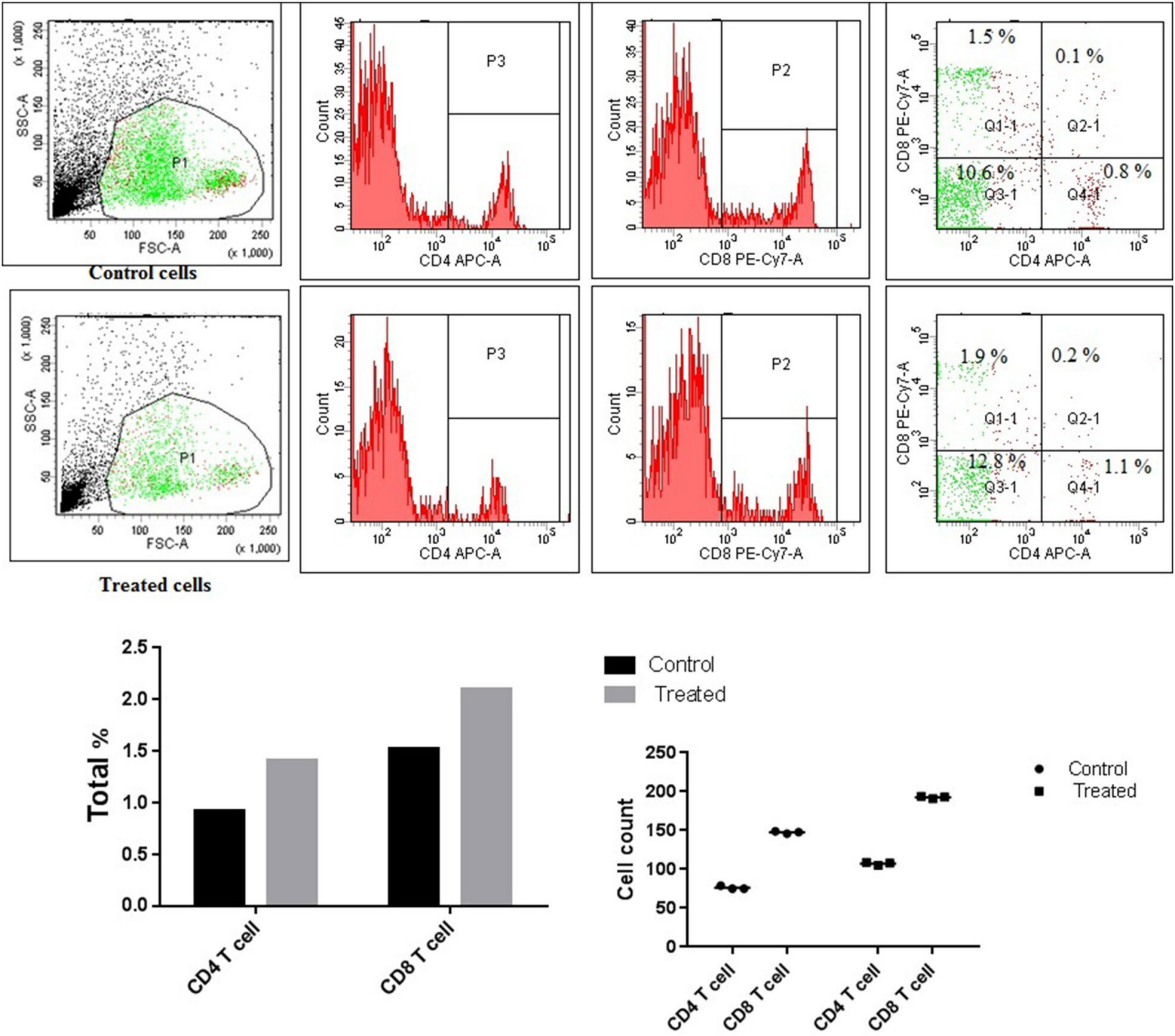
Effects of purified pentasaccharide (LA-EPS-20079) on the CD4 and CD8 T cells expression of PBMC

**Fig. 8:**
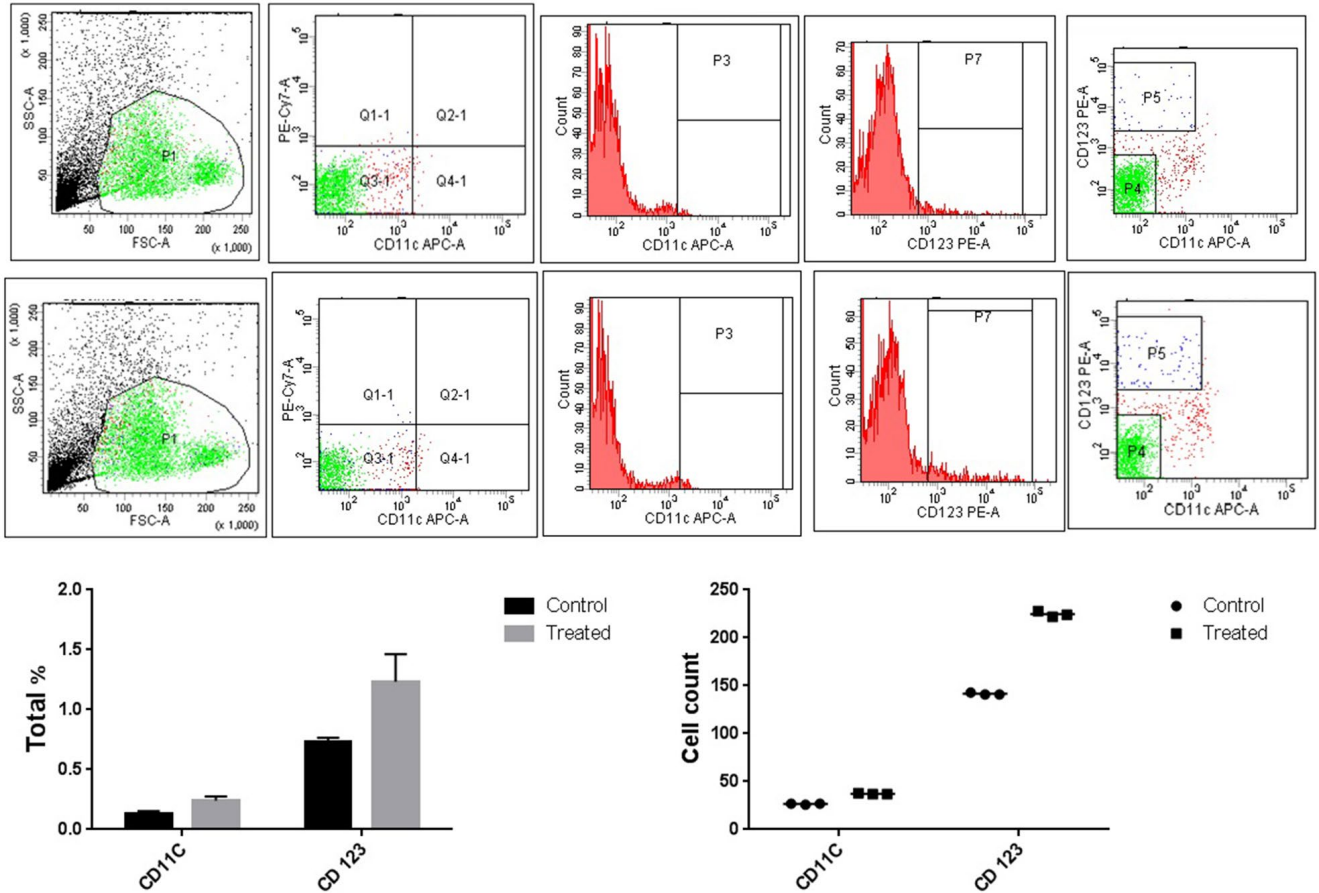
Effects of purified pentasaccharide (LA-EPS-20079) on the CD11b and CD123 dendritic cells expression of PBMC

At least 2 × 10^4^ cells were analyzed using flow cytometry after treatment with the non-toxic concentration of the extract for 72 h. Lymphocytes were gated in the forward side scatter plot and the frequency of T cells was detected. Within the T cell populations, CD8+ T (cytotoxic T cells) cells and CD4+ T (T helper cells) cells frequency was determined. *L. acidiophilus* LA-EPS-20079 showed to increase the percentages of CD4+ (0.95% ± 0.01% in control vs. 1.4% ± 0.1% after 72 h at 2.61 mg/ml, p = 0.01), and CD8 + T cells (1.5% ± 0.1% vs. 2.1% ± 0.3%, p = 0.001) (Fig. 7). On the other hand and Within the dendritic cells (DC) populations *L acidiophilus* LA-EPS-20079 showed to increase the percentages of CD11b (0.1% ± 0.01% in control vs. 0.2% ± 0.03% after 72 h at 2.61 mg/ml, p = 0.01) and CD123 (0.7% ± 0.01% vs. 1.1% ± 0.2%, p = 0.001) (Fig. 8).

#### Induction of intracellular ROS using *L. acidophilus* EPS in both cancer and normal cells

**Fig. 9:**
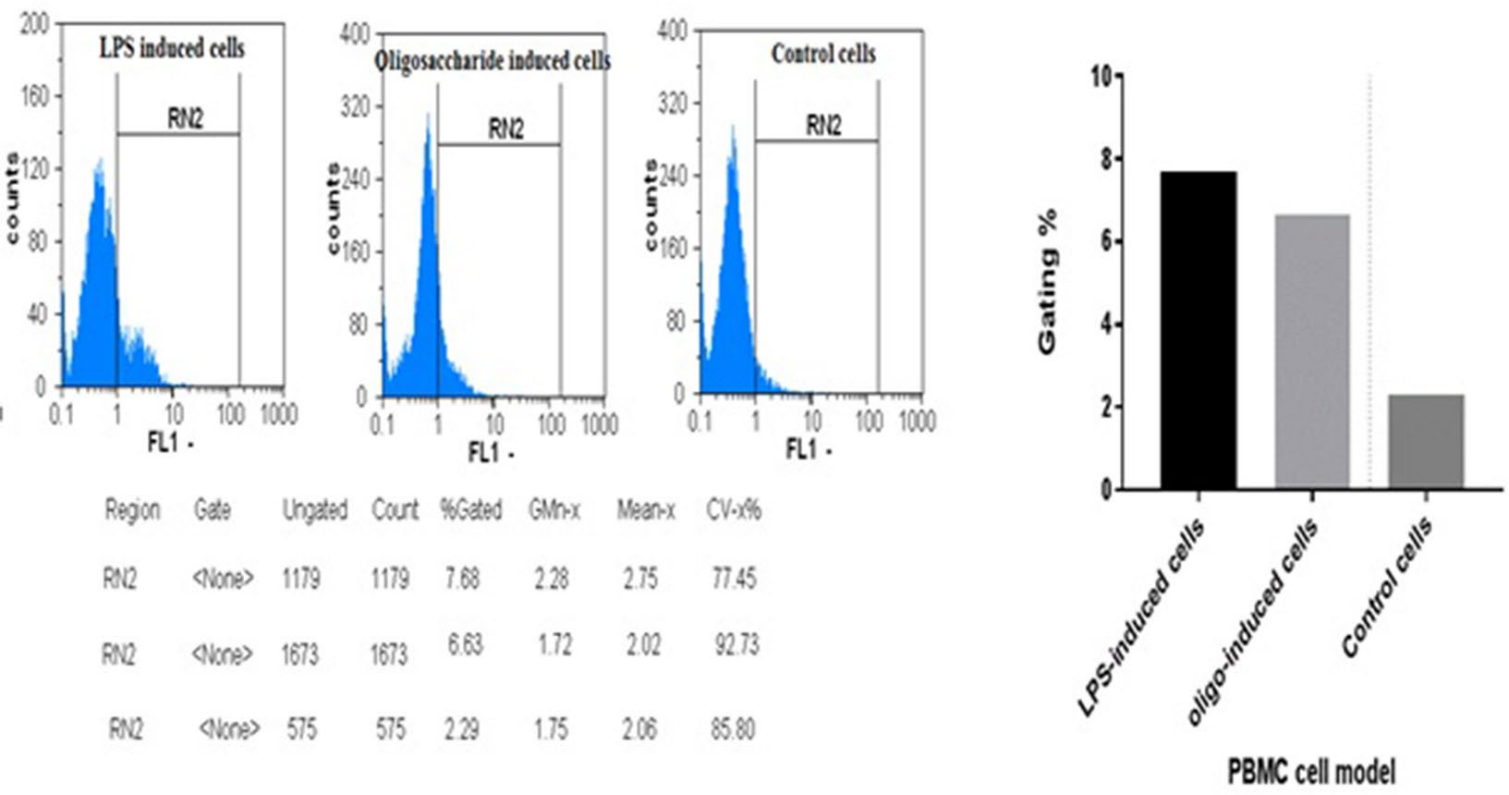
Quantification of total induced ROS in CaCo-2 and normal MRC5 cells treated with purified pentasaccharide (LA-EPS-20079) using flow cytometry

The induced intracellular reactive oxygen species (ROS), in both colon cancer and normal epithelial cells after treatments with EPS were quantified by measuring the emitted relative fluorescence upon the oxidation of the reduced form of the fluorescence probe (DFCH), by ROS, using the flow cytometry. The obtained data in Fig. 9 showed, generally, a dramatically increase in the gating percentage of dichlorodihydrofluorescein (DCF) fluorescence in CaCo-2-treated cells (from 4.05 to 20.28%), comparing with the non-treated cells. In contrast to the normal epithelial MRC5 cells, after treatment, the emitted fluorescence gating percentage was changed slightly without any significant differences from the non-treated cells (3.51–2.05%).

#### Quantification of the induced cytokines and ROS in LPS-induced PBMCs cell model

**Fig. 10:**
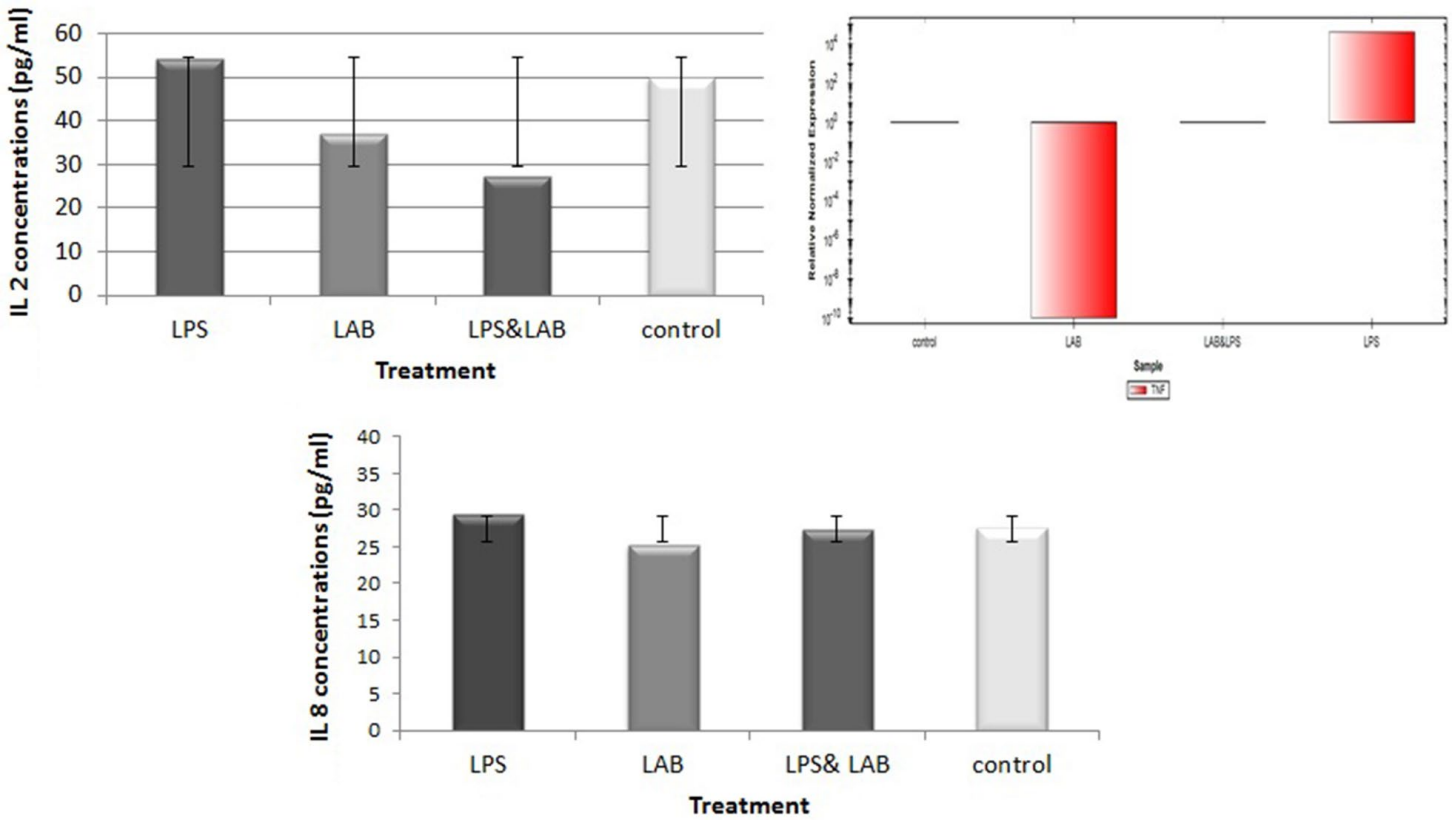
Quantification of total induced ROS in PBMC models using flow cytometry

Using flow cytometry, *L. acidophilus* polysaccharides, showed the ability to induce intracellular ROS in PBMC model with gating value raised from 2.29% (in control cells), to 6.63% (in treated cells), but, still with smaller value than that of LPS-induced cells (7.68%) (Fig. 10).

**Fig. 11:**
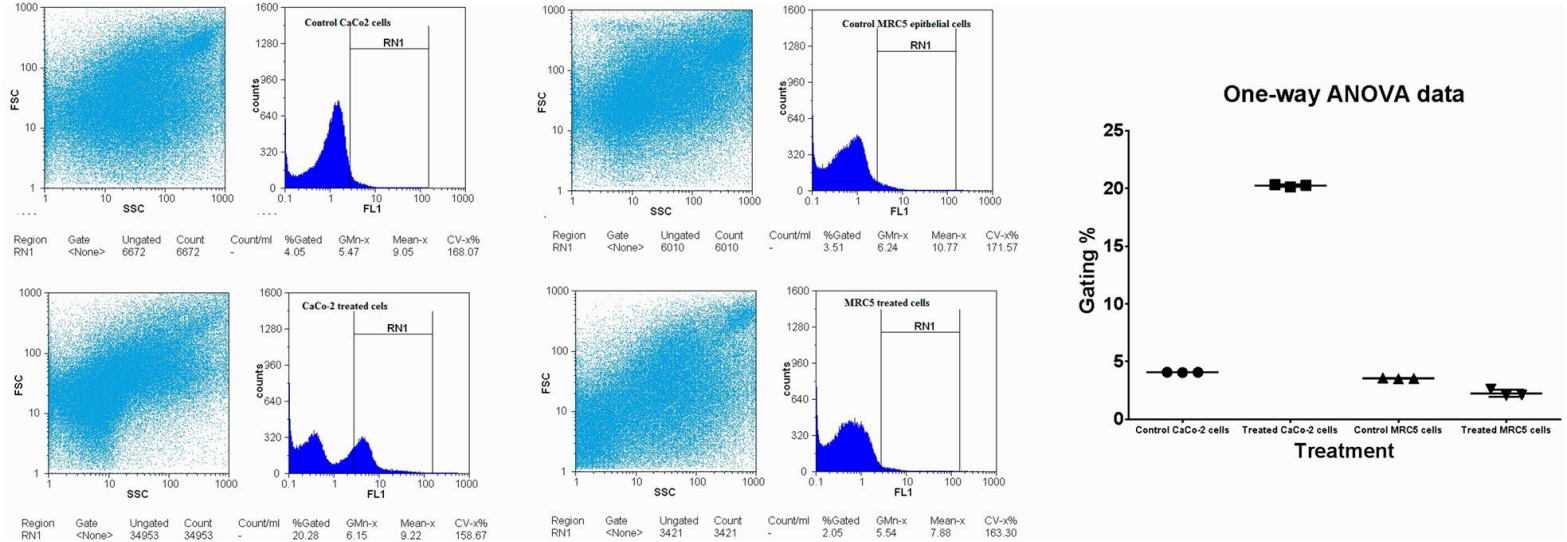
IL2&8 concentrations and TNF-α gene expression levels in PBMC incubated with the purified pentasaccharide (LA-EPS-20079)

On the other hand, in LPS-induced PBMCs cell model and in comparison with the control non-treated cells, the recovered LA-EPS-20079, reduced the concentration of the induced IL-8 from 27.6 to 25.27 pg/ml (Fig. 11). Also, after treatment, the level of IL-2 decreased from 54.2 to 27.07 pg/ml in LPS-induced cells (Fig. 11) with reduction in the expression level of the TNF-α gene to its normal state (Fig. 9).

#### Regulatory effect of *L. acidophilus* LA-EPS-20079 on NF-κB inflammatory pathway

The expressions of TGF, IKaB and P53 genes, as a response to the treatment were quantified using RTqPCR. Upon normalization with β-actin gene, the expressions of P53 and IKaB genes were up-regulated during treatment. The anticancer activities of *L. acidophilus* LA-EPS-20079 via NFκB inflammatory pathway could be explained by the reduction in IL8 levels, with up regulation in the expression of IKaB gene (Table 3).

**Table 3:** ^1^H and ^13^C NMR chemical shifts (ppm) at 70 °C of the resonances of LA-EPS-20079

Sugar residue	^1^H/^13^C							
		1	2	3	4	5	6	6
→2) α-d-GlcA(1→	A	92.18 5.21	76.03 3.63	71.99 3.80	72.85 3.55	72.15 4.18	176.19	
→2) α-d-GlcA(1→	B	96.00 5.14	75.84 3.67	71.53 3.91	72.16 3.56	72.62 4.18	176.15	
→2,4) α-d-GlcA(1→	C	95.39 5.08	76.03 3.73	70.36 4.12	77.96 3.73	71.58 4.18	174.12	
α-l-Fuc (1→	E	100.28 4.55	68.99 3.78	69.96 3.80	72.52 3.78	68.05 4.19	16.24 1.27	
α-d-Glc (1→	D	96.00 4.68	72.15 3.53	74.28 3.73	71.29 3.43	72.85 3.99	62.22 3.73	3.83

## Discussion

Cancer cells are known to develop chemotherapy resistance throughout the course of treatment. Thus, there is a continuously growing demand for novel anti-cancer agents Ferlay et al. [1]. In this study, we have succeeded in the extraction, purification and characterization of a novel pentasaccharide LA-EPS-20079 from *Lactobacillus acidophilus* with selective cytotoxicity toward CaCO-2 cell line compared to non-cancerous cells. The structural analysis of LA-EPS-20079 was performed using the ‘determine structure’ module of the CASPER program Jansson et al. [18]; Lundborg and Widmalm [19] and Rönnols et al. [20], suggesting the following structure residues; three d-GlcAp, one l-fucose and one d-Glc residues. Furthermore, previously published ^1^H-and ^13^C-NMR data by Dutton and Merrifield [21]; Cescutti et al. [22]; Wang and Yang [23]; Perry et al. [24] and Fotakis and Timbrell [25], together, with our current complete assignment of ^1^H and ^13^C chemical shifts and the inter-residue connectivity, of the monosaccharides residues of LA-EPS-20079 allowed LA-EPS-20079 to be formulated as α-d-Glc(1→2)][α-l-Fuc(1→4)] α-d-GlcA(1→2) α-d-GlcA(1→2) α-d-GlcA.

The majority of anti-cancerous agents were found to be toxic to normal cells, making the discovery of new safer compounds an important demand in the biomedical field [25]. Lactic acid bacteria possess GRAS (generally regarded as safe) status allowing their use without labeling. Neutral red assay is considered as one of the most sensitive tests, used for evaluating the cytotoxicity exerted on various cancer cell lines based on the accumulation of the neutral red dye within the lysosomes of the intact healthy cells as reported by Fautz et al. [26] and Nami et al. [27]. In the present investigation, cytotoxicity assay data, revealed the presence of non-identical cellular responses to LA-EPS-200790 treatment depending on different cell death mechanisms and their sensitivity to the treatment. For more details, our recorded IC50 doses of the polysaccharide on normal mammalian cells, ranged from 2.61 mg/ml to 0.68 µg/ml. MDCK were found to be the most sensitive cell lines to EPS, with IC50 2.61 mg/ml, while VERO and MRC5 were the most tolerant ones. Almost the same finding was reported by Nami et al. [27], where they found that *E. lactis* IW5 secretions had no toxic effect on healthy cells, with 95% of the cells grew normally.

In vitro cell models play an important role in understanding cellular related to up normal human physiological conditions. García-Lorenzo et al. [28] reported an upregulation in non-differentiated Caco-2 cells, especially, in proteins involved in cell growth and proliferation related to cancer, confirming the tumoral phenotype of these cells. To that end, investigating the anti-cancer effect of *L. acidophilus* was performed with verification of the probable effect of its polysaccharides extract on the inhibition of colon cancer cell proliferation (87.27%) and cell death (80.65%) with 1.96–51.3 colon cancer selectivity index. The used pentasaccharide showed a strong cancer cell selectivity index, reached 51.3, suggesting that the inhibitory effects of *L. acidophilus* LA-EPS-20079 on colon cancer cells proliferations could be accompanied by an increase in the proliferation of non-cancerous cell, Kahouli et al. [29] have demonstrated similar findings, where *L. fermentum* showed inhibition activities against colon cancer cells with increasing proliferating rate of non-cancerous colon cell growth. They reported that, the extracts of *L. fermentum* NCIMB 5221*, L. rhamnosus* ATCC 53103, and *L. acidophilus* ATCC 314, could show abilities to inhibit colon cancer cells with promoting the proliferation of non-cancerous epithelium [29]. Also, they discussed that, the selectivity effects of the used lactic acid bacterial strains on cancer cells could be attributed to the presence of propionate and butyrate (the end products of the fermentation); they consider as histone deacetylases (HDACs) inhibitors that induce differentiation in normal colon cells but cause apoptosis in colon cancer cells [29].

The complete analysis of sugar moieties helped us to explain the possible mode of action of the extracted LA-EPS-20079 pentasaccharide. The spectroscopic analysis of LA-EPS-20079 indicated the presence of 3 glucuronic acid moieties, with one fucose and one glucose moieties. Based on our findings of the current research and the previously reported research studies; the anticancer activities of LA-EPS-20079, could be attributed to the presence of glucuronic acid and fucose moieties in its structure. Choy et al. [30], showed strong antitumor activity of low molecular weight polysaccharide (3 Kd; composed partly of uronic acid), extracted from Angelica Sinensis rhizome, with immunostimulating activities in both in vitro and in vivo levels. Also, El-Nezhawy et al. [31], chemically synthesized a series of d-glucuronic acid derivatives, starting from the d-glucuronic acid, with positive in vitro antitumor activities against MCF-7, TK-10 and UACC-cell lines. Furthermore, few multiple reports, explained the possible anticancer activity of l-fucose moieties. For example, Wolfe et al. [32], reported a direct action of l-fucose against tumor cell in vitro with a complete growth inhibition of the cancer cells, at a concentration of 50 mg/ml and 60% inhibition, at a concentration of 12.5 mg l-fucose per milliliter. On the other hand, l-fucose showed an indirect antitumor activity, as illustrated for both crude and purified fucoidans, extracted from *Sargassum cristaefolium,* against HT-29 human colon cancer cells causing G1 phase cell arrest Wang et al. [33].

At molecular level, apoptosis occurs through two main pathways; intrinsic and extrinsic. Intrinsic or mitochondrial pathway is mediated by Bcl-2 family of pro- and anti-apoptotic proteins. The extrinsic apoptosis pathway or cytoplasmic pathway; is mediated through the Fas death receptor, a member of the tumor necrosis factor [34]. The tumor suppressor P53 may be involved in the regulation of pro-apoptotic genes associated with both intrinsic and extrinsic pathways [35]. The effect of p53 in regulation of tumor cells apoptosis via regulating NF-κB activity has been demonstrated in several studies. The phosphorylated p53 interacts directly with IKKβ resulting in reduced IκBα-phosphorylation as a consequence, nuclear translocation of NF-κB is inhibited [36]. The results of the current study assume that upon treatment; the activation of p53 is the responsible mechanism for NF-κB inhibition via up-regulating IκBα expression and subsequent confirmed the clear cut interaction among p53, NFκB and apoptosis in colon cancer-treated cells. In addition, transforming growth factor-β (TGF-β) is a multifunctional polypeptide that can be switched from a tumor suppressor, in normal, or dysplastic cells, to a tumor promoter in advanced cancers. Although TGF-β is a prominent tumor suppressor in most early cancer cases, it could show abilities to enhance the non-epithelial tumor proliferation, invasion, metastasis, and angiogenesis in this established tumor [37, 38]. In addition, multiple reports indicated that, TGFβ–dependent induction of epithelial-mesenchymal transition (EMT), in late-tumorigenesis stage counted, at least in part, on the activity of NF-κB pathway [39]. Interestingly, upon treatment of colon cancer cells with *L. acidophilus* LA-EPS-20079, an up regulatory effect of the pentasaccharide was detected, on TGF gene with down regulatory action on IκBα. This finding, with the above-mentioned hypothesis, raised a question about the possible crosstalk between these two signalling pathways. This suggests that, during cellular treatment with *L. acidophilus* polysaccharide pentasaccharide, TGF-β, retrieved its own character in normal epithelial cells as a potent tumor suppressor through apoptosis stimulation. Additionally, we could hypothesise that, LA-EPS-20079 treatment counteracted the aggressive behaviour of cancer cell to attenuate the apoptotic canonical TGF-β-SMAD pathway and activated non-canonical TGF-β-NF-κB inflammatory signalling pathways. Our findings could be supported by the findings of Sovak et al. [40], they indicated, in certain breast cancer cell lines, that TGF-β showed capabilities of suppressing NF-κB signalling through up-regulating the expression of IκBα and subsequently inhibit the NF-κB pathway.

Among all the NF-κB activators, tumor necrosis factor (TNF-α) is considered the most potent cytokine and its activated pathway is well understood [41]. In response to TNFα and cytokines binding to its receptors, TGF-β-activated kinase 1, directly phosphorylates the inhibitor-kappaB kinase (IKK) complex that promotes the activation of NF-κB [42]. Also, IL-2 receptor signalling activates Phosphatidylinositol 3-kinase that in turn stimulates the NF-κB activity by up-regulating I-κB degradation [43]. Furthermore, the activation of NF-κB pathway is considered as one of the most significant factors that generate maximal IL-8 amounts, beside JNK pathways activation and the depression of the gene promoter factors [44]. The positive effects of lactic acid bacteria on cytokine production are complex and inconsistent. Some strains appear to enhance the anti-inflammatory cytokine production, interleukin (IL)-10, while having no effect or slightly decreasing production of the proinflammatory cytokines; chemokine, IFN-γ and TNF-α [45]. In addition, Studies on the effect of lactic acid bacteria on cellular immunity are very limited. Our data indicated that EPS stimulated CD 123 and CD11b expression by CD4+ andCD8+ T lymphocytes. Similar studies that indicated the same findings have been reported in a few cases. For example, Castellazzi et al. [46] demonstrated that *L. paracasei* I 1688, *L. salivarius* I 1794 increased the percentage of CD4+/CD25+ cells (T helper-activated regulatory T cells), CD8+/CD25+ (T suppressor/cytotoxic-activated cells) and CD16+/CD56+ (NK cells) (p < 0.05). So we can conclude that *Lactobacillus acidophilus* DSMZ 20079 cell-free pentasaccharide enhances immune function, particularly that of cytotoxic T lymphocytes (CD8+ T cells) and through the inhibition of TNF, IL2 and IL8 cell-signalling pathways by our treatment could be another factor that suppresses the NF-κB pathway and subsequently, prevent tumor cells proliferation. This was accompanied by the production of IFN-γ, interleukin-β (IL-1β) and TNF-α inhibiting tumor growth [47].

In conclusion, our study evaluated the anticancer activity of *L. acidophilus* LA-EPS-20079 pentasaccharide by testing its direct antitumor effects on different cancer cell lines, as well as, its immunomodulatory effects. This study suggested that, the newly identified *L. acidophilus* EPS exerted a direct cytotoxic action on the tumors cells via apoptotic mechanisms in addition to stimulating the immune response and inactivating NF-κB inflammatory pathway, providing novel targets to the current therapeutic manipulation of cancer. Finally, based on the current evidences, the effects of *L. acidophilus* EPS on colon cancer is considered to be very promising, although, in vivo studies are required to identify the possible interactions between the bacterial EPS and the host immune system to ensure its efficacy and safety in the prevention or treatment of colon cancer.

## Additional file

**Additional file 1: Figure S1.** Growth curve.
