# Supplementary figures and images for: A novel purified Lactobacillus acidophilus 20079 exopolysaccharide, LA-EPS-20079, molecularly regulates both apoptotic and NF-κB inflammatory pathways in human colon cancer
Source: Microb Cell Fact. 2018 Feb 21;17:29. doi: 10.1186/s12934-018-0877-z (PMC5820793; doi:10.1186/s12934-018-0877-z)

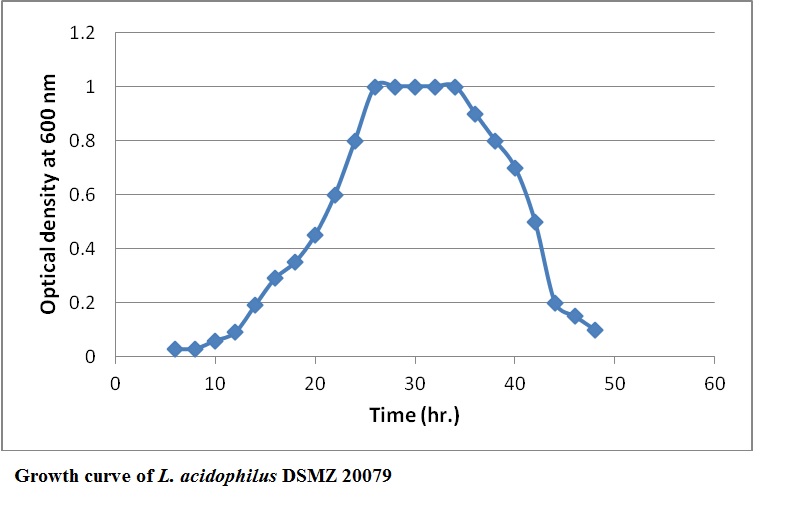

Supplement: Supplementary file 1 — Additional file 1: Figure S1. Growth curve. [file 12934_2018_877_MOESM1_ESM.jpg]
